# Healthcare in Vietnam: Harnessing Artificial Intelligence and Robotics to Improve Patient Care Outcomes

**DOI:** 10.7759/cureus.45006

**Published:** 2023-09-11

**Authors:** Tu N Doan Thu, Quan K Nguyen, Andrew W Taylor-Robinson

**Affiliations:** 1 Business and Management, College of Business and Management, VinUniversity, Hanoi, VNM; 2 Epidemiology and Public Health, College of Health Sciences, VinUniversity, Hanoi, VNM; 3 Epidemiology and Public Health, Center for Global Health, University of Pennsylvania Perelman School of Medicine, Philadelphia, USA; 4 Epidemiology and Public Health, Smart Health Center, VinUniversity, Hanoi, VNM

**Keywords:** ai & robotics in healthcare, surgery, robotics, artificial intelligence, digital technology, vietnam, healthcare

## Abstract

Healthcare in Vietnam is increasingly utilizing artificial intelligence (AI) and robotics to enhance patient care outcomes. The Vietnamese healthcare sector recognizes the potential of AI and is actively exploring its applications in research and clinical practice. AI technologies, such as text mining and machine learning, can be employed to analyze medical data and improve decision-making processes. Robotics, on the other hand, can support various healthcare tasks, including elderly care, rehabilitation, and surgical interventions. Robotic surgery, specifically, is an innovative form of minimally invasive surgery that aims to improve surgical outcomes and enhance the patient experience. The implementation of AI in emergency and trauma settings is still in its early stages, but there is a growing interest in and recognition of its potential benefits. However, there are challenges that need to be addressed, such as the need for appropriate research and training programs to support the adoption and integration of AI in healthcare. Despite these challenges, healthcare professionals in Vietnam are optimistic about the potential of AI to improve acute care surgery and are open to embracing new digital technologies. The use of AI and robotics in healthcare aligns with the broader goal of improving healthcare systems in low- and middle-income countries, including Vietnam, through technological advancements. Overall, AI can play an important role in assisting prognosis and predictive analysis by integrating vast amounts of data. Moreover, the integration of AI and robotics in healthcare in Vietnam has the potential to enhance patient care outcomes, improve decision-making processes, and support healthcare professionals in their practice.

## Editorial

Introduction

The recent emergence and rapid convergence of digital technology with the healthcare industry has come to define a global environment in which artificial intelligence (AI) and robotics not only leverage medical practice but also improve patient outcomes and safety. AI has been integrated into domains that were formerly reserved for human expertise [1], and the healthcare landscape now groups AI applications into four overarching categories: expressive; analytical; prognostic; and prescriptive [2]. The impact of AI has multiplied exponentially across numerous subfields, including fundamental biomedical sciences, translational medical research, and clinical practice [1, 3-5]. The utilization of AI-assisted tools in exacting domains like image-based diagnostics and genome interpretation is tackling effectively the challenges posed by vast data volumes, significantly enhancing their efficacy. Notably, AI offers the capacity to strengthen the healthcare industry by assuring individualized patient care, establishing secure workplace environments for healthcare providers, and maximizing the operational efficiency of hospitals and clinics, be they in the public or private sector [2].

The global landscape of AI also showcases the expansion of robots in healthcare. The response to the COVID-19 pandemic spurred the integration of robots in surgical environments, addressing unique challenges and enhancing safety protocols [6]. For instance, a recently published randomized clinical trial reveals that robot-assisted radical cystectomy significantly increased the duration of patient survival outside the hospital among non-metastatic bladder cancer cases [7]. Beyond the operating theater, robotics is becoming integral to clinical settings, providing support to healthcare professionals and elevating the quality of patient care. The reach of robotics and automation extends into research laboratories, automating repetitive tasks and enabling scientists and technicians to dedicate more time to strategic endeavors, thereby accelerating discovery processes. It may be argued, however, that these applications are too distant from routine clinical practice for their streamlining to have a significant translational impact. Amid these advancements, considerations arise regarding the ethics of caregiving by robots and public acceptance of automation of regulated aspects of health care delivery. This underscores the pivotal need to harmonize technological progress with human comfort and trust, as well as to weigh up the cost-effectiveness and potential impact of incorporating robotics into various medical fields.

Vietnam - on the cusp of a digital health transformation

**Figure 1:**
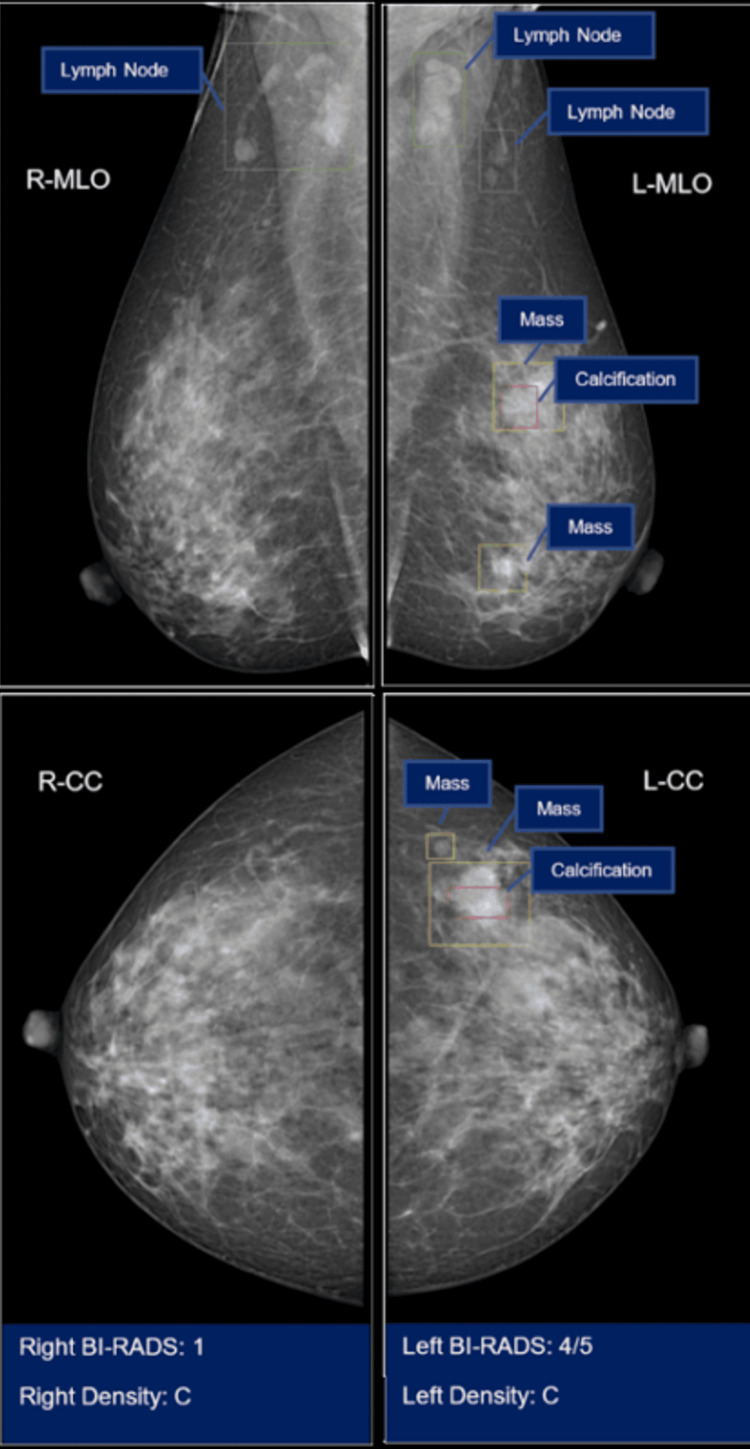
Breast imaging-reporting and data system (BI-RADS) labeling with VinDr AI. Courtesy of VinDr AI at https://vindr.ai; accessed September 7, 2023.

Following this global trend, Vietnam's healthcare system is progressively adopting AI and digital health technology applications, in particular in pathology, albeit at a slower rate than in some other nations [8]. Within the next 10 years, computational and integrative pathology will experience tremendous growth that will improve diagnosis, prognosis, and prediction. Currently, however, digital readiness in the country is still relatively low, and the implementation of digital health technologies remains in its infancy [9]. According to an analysis conducted last year, the projected AI adoption rate for Vietnam is 9%, the lowest among those nations surveyed, and well below the global average of 23% deployment [10]. Yet, numerous entities are developing AI models that can detect diseases such as COVID-19, tuberculosis, pneumonia, and breast cancer by screening medical images [11]. The Big Data Research Institute at VinBigData has developed ‘VinDr’, an AI-powered system for analyzing chest X-rays, mammograms, and genitourinary tract ultrasound or computerized tomography scans to detect lung diseases and prospective breast, testicular, and prostate cancers (Figure 1). The system has been rigorously evaluated by renowned tertiary care centers in Vietnam, including the 108 Military Central, Hanoi Medical University, and Vinmec Healthcare System [12].

For the VinDr AI system, open-source data can be accessed through the link https://lab.vindr.ai, an online repository where data of medical images including X-rays of the chest, spine, and ribs are readily accessible to the healthcare community (Figure 1). Visitors can view labeled images from the datasets, thereby validating the dataset’s image quality and reliability with their own accounts. In this context, it is relevant to note that AI-enhanced analysis improves the rate of detection of abnormalities at very early stages and its applications are far beyond reading X-ray, CT-scan, or MRI images, as AI can integrate data from a vast quantity of pathological specimen slides, time-series data from wearable devices, or clinical records in general, to aid in diagnosis, prognosis, and administration of predictive medicine. This provides impressive precision, enhancing diagnostic accuracy and lowering the proportion of false negative results through combining clinical and preclinical data with consideration of a wide range of variables.

**Figure 2:**
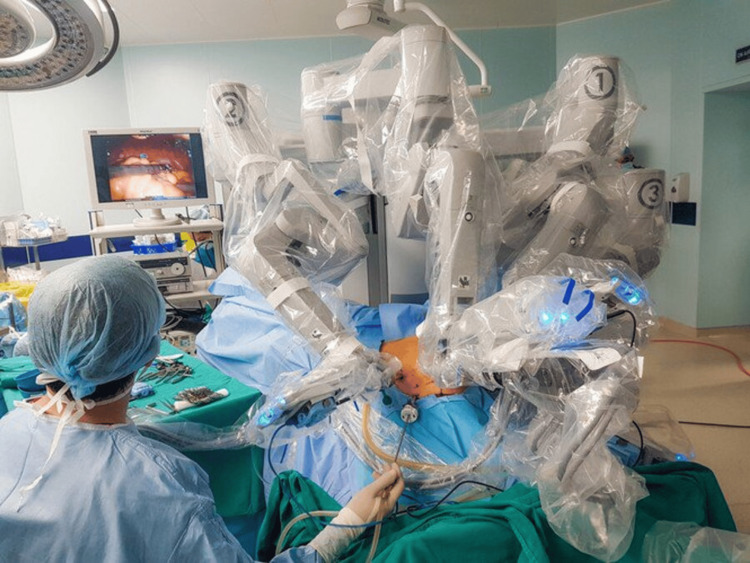
da Vinci Surgical System in use at the Vinmec Healthcare System. Courtesy of Vinmec at https://www.vinmec.com; accessed September 7, 2023.

Vietnam's strides in healthcare technology are evident from the establishment of a pioneering robotic surgery section at Binh Dan, a leading public hospital in Ho Chi Minh City, in December 2016. This milestone marked a significant breakthrough in advanced patient care as it represents Vietnam's first authorized use of surgical robots on adults [13]. This trend in surgery has since spread to other major public hospitals, including the National Children's Hospital, Bach Mai, Viet Duc, and Cho Ray, which have each trialed or introduced robotic surgery initiatives. Furthermore, the country’s first privately funded robotic surgery center was opened at the Vinmec Healthcare System in July 2018 [14]. Remarkably, these institutions all make extensive use of the minimally invasive da Vinci Surgical System (Figure 2), which is typically confined to advanced medical centers in high-income nations. The da Vinci robot detects the surgeon's hand movements that it translates into micro-scaled motions to manipulate the miniature proprietary instruments. Notably, the system’s software also detects and filters out any tremor-like movements by the surgeon's hand and so impedes direct replication by the robot arm, leading to enhanced precision. The Versius Surgical Robotic System, which was introduced in 2019, is now a major competitor to the da Vinci robotic technology. It claims to be more flexible and versatile, with "quick and easy to set up" modular arms. Although both of these examples are significant enterprises that make a persuasive case for adopting robotic surgery in Vietnam, its widespread uptake will depend on substantial government support. In line with this progression, this article delves into the present challenges, opportunities, and future implications of AI and robotics in Vietnam's digital healthcare landscape.

Artificial intelligence in healthcare

A Promising Start

The contemporary AI landscape in Vietnam is shaped by several compelling factors. By introducing policies and funding initiatives, the socialist government has demonstrated strong support and commitment to the development and incorporation of digital health solutions. This leadership is underscored by action plans such as Decision No. 749/QD-TTg [15], which positions healthcare as a central element of the national digital transformation strategy. The implementation of Decision No. 5349/QD-BYT, which calls for an electronic health records master plan, together with the focus of Decision No. 4888 on smart health, further highlights the nation’s vision to harness AI and digital healthcare [16, 17]. The rapid rise and deep penetration of the internet, mobile smart devices, and cloud technologies provide the infrastructure and platforms required for the seamless distribution and accelerated access to digital health innovations [18, 19]. This digital infrastructure, alongside regulatory frameworks, constitutes the backbone of AI deployment, enabling real-time data gathering, analysis, and dissemination, thereby boosting the prospect of enhanced healthcare solutions.

Changing consumer preferences and healthcare provider needs have paved the way for the incorporation of AI-driven solutions. The global health turmoil caused by the COVID-19 pandemic has greatly raised awareness and expedited the use of digital health solutions [20], thereby inadvertently creating a setting conducive for AI-based innovations to thrive. Online consultations, telemedicine services, e-prescriptions, e-commerce platforms, and remote monitoring technologies have all increased as a result. The diversity and availability of domestic and international digital health companies, investors, and strategic partners also help usher in AI and digital healthcare in Vietnam [21, 22]. The emerging ecosystem of stakeholders who provide considerable knowledge, creativity, and resources to the development and distribution of AI-based solutions throughout the healthcare system elevates Vietnam's AI trajectory. A promising route for expansion is tapping the tremendous potential for collaboration and engagement with international organizations entrenched in AI and digital health expertise. Vietnam may escalate its rate and scale of AI-driven healthcare innovation by adapting the knowledge and enhancing the experience of these foreign institutions to the local context. In this way, the country aims to provide a tailored, sophisticated, and effective healthcare delivery.

Challenges Lie Ahead

The global landscape of AI in healthcare also presents a complex tapestry of challenges. Despite the current legal frameworks, progress is hindered by the lack of comprehensive standardized guidelines for the international development and deployment of digital health technologies [23]. In addition, inadequate infrastructure and human resources in rural and remote locations impede access to digital health applications, which compounds deep-rooted social issues of regional inequity [24]. Moreover, the rigid customs of minority ethnic groups in rural areas will likely lead to hesitancy in accepting AI robotics. The possible lack of interoperability and integration between disparate digital health platforms and data sources impedes the exchange of information. The skepticism of both the general public and healthcare professionals regarding the benefits and security of digital health technologies contributes to low levels of awareness and trust. Additionally, concerns about data privacy, consent, equity, and the accuracy of data interpretation are among the ethical and social implications of digital health adoption [25]. Navigating these multiple complexities is essential for AI to be incorporated effectively and ethically into global healthcare systems.

The implications to Vietnam of embedding AI in healthcare include crucial domains that are destined to revolutionize the sector. Currently, AI is applied in four major areas, namely disease diagnosis, treatment, health monitoring information management, and staff training [26]. Integrating remote patient monitoring using AI-driven tools, wearable devices, and sensors to measure vital health parameters such as blood glucose concentration, pressure, and oxygen saturation has transformative potential. Virtual AI-support tools and remote monitoring capabilities are poised to enhance telemedicine, thereby optimizing patient care in the community. The Smart Health Center, a research collaboration between the Hanoi-based VinUniversity and the University of Illinois at Urbana-Champaign, is making encouraging progress in this field [27]. As the nation looks to the future, other promising research areas emerge: (i) scientific data mining and compilation, ranging from medical records to comprehensive data repositories; (ii) comprehensive reports involving clinical, paraclinical, and medical services; and (iii) a prolonged focus on biomedical studies, encompassing traditional clinical inquiries based on genotypes and clinical profiles. In order to ensure the prudent integration of AI, continuous revision of the legal framework is essential. The maxim from computer science of “garbage in, garbage out” in relation to flawed data means that effective algorithm development necessitates maintaining high-quality data input [25]. In addition, in a medical context ethics are a major concern, guiding the deployment of AI models while upholding ethical principles, particularly those involving human participants.

Robotics in healthcare

The Surgical Revolution

The implications of robotics for the future of healthcare in Vietnam are promising, particularly in surgical procedures [28]. Among numerous disciplines, including geriatric care and rehabilitation, surgery stands out as a prominent application of robotics, presenting opportunities for improved outcomes. Robotics is starting to revolutionize surgical practices by minimizing invasiveness, trauma, and hemorrhage. This has the knock-on benefit of substantially reducing the risk of post-operative infections. It also helps to minimize hand tremors for surgeons, which was hitherto an occupational hazard when a procedure required great manual dexterity and precision over an extended time. Moreover, the robot arm's flexible joints enable extraordinary wrist rotations of 180 degrees, a feat that is impossible for humans or conventional laparoscopic instruments. This adaptability further enhances surgical precision by facilitating precise access to intricate regions of the body. The surgical precision enabled by 3-D imaging viewed through a 12x magnifying camera lens is comparable to that of open surgery. The combination of rotary joint instruments and a wide surgical angle of 540-580 degrees simplifies complex laparoscopic procedures [29]. As the surgeon’s recovery is expedited, complications arising from fatigue are minimized. Also, the robot's computer connectivity enables remote surgery, which transcends geographical limitations. Depending on the financial viability of utilization in such scenarios, this capability is especially valuable in remote and disaster-stricken regions. As robotics continues to advance, these innovations are expected to reshape the surgical landscape in Vietnam, enhancing patient care and promoting operating excellence.

Obstacles to Implementation

Notwithstanding the outlined prospects for advancement, the implementation of robotic surgery in Vietnam faces a number of obstacles that hinder its widespread adoption and effectiveness. The potential cost, met in full or in part by the patient, of thousands of dollars per procedure is one of the greatest hurdles. This financial burden is particularly significant in Vietnam, where the average annual income is comparable to the cost of a single robotic operation. In addition, surgical procedures such as thyroidectomy that are completed relatively quickly when performed by conventional manual techniques take significantly longer when conducted robotically. Hence, an operation that would ordinarily take 30 minutes at a central endocrinology hospital can take up to two hours. The proficiency required by surgeons to perform such a procedure successfully is a further significant impediment. Transitioning from a traditional surgical background to becoming accomplished in robotic techniques requires highly specific training. A surgeon must acquire a new skill set and amend their experienced routine, which requires a positive attitude towards continuing professional development as well as a substantial investment of both their time and resources. In the interest of addressing these issues, concerted efforts are required to train healthcare professionals and advocate for the accessibility and affordability of robotic surgery in the Vietnamese healthcare environment [30].

While challenges like installation costs, safety concerns, and integration issues have been identified as barriers to the widespread adoption of robotic surgery, the field's trajectory is upward. The expectation of a future scale-up of surgical robotics implies a tipping point will be reached when system costs and the time involved are reduced to manageable levels. As technology evolves and becomes more refined, it is anticipated that the cost-effectiveness of robotic surgery systems will increase, leading to greater market penetration and accessibility. This change not only bodes well for the healthcare landscape in Vietnam but also suggests a brighter future globally in which those in need will have easier access to cutting-edge medical interventions. Novel solutions may emerge, such as the machine hire scheme at Ho Chi Minh City’s 115 People’s Hospital, which is supported by a transparent public bidding process [31]. By adopting this non-purchase strategy, the otherwise prohibitively high initial investment could potentially be overcome, thereby making robotic surgery more accessible to a broader range of medical facilities.

Financial Considerations

Achieving comprehensive healthcare coverage in Vietnam remains an aspirational goal that necessitates public access to an affordable health insurance framework. The government should enact insurance policies that strike a delicate balance between cost-effectiveness and recouping the outlay on autonomous surgery. A crucial aspect of this scheme would be the creation of a ‘targeted disease list’ that prioritizes conditions requiring precise surgical interventions that would be enhanced by AI robotics. For instance, complex neurosurgery, removal of various malignancies, and coronary artery bypass grafting should all be at the top of the insurance coverage agenda, enabling patients who would otherwise face financial barriers to receive advanced treatments. Additionally, the specialist field of pediatric robotic surgery merits consideration within the insurance support framework. Common childhood conditions such as bile duct cysts, congenital megacolon, and specific intrathoracic procedures are sufficiently significant to warrant inclusion [30]. This all-inclusive approach to insurance coverage is essential to ensuring that the benefits of robotic surgery are accessible to all population groups to which a person may belong, regardless of sociodemographic profile or medical condition. Given that an impressively high 89.3% of eligible Vietnamese adults currently have health insurance (albeit typically a more basic policy), a figure that is projected to rise to 95% by 2025, this strategy may work effectively in the context of this country [32].

Conclusions

The healthcare industry has historically relied on manual processes that can be time-consuming and prone to human error. However, recent advances in machine learning are set to significantly alter the environment, the combination of AI and robotics heralding a new era of digitalized healthcare provision. It is envisaged that these technologies will be increasingly incorporated into routine workflows, thereby helping healthcare professionals to improve quality and address consistency issues. Yet, such automation does not replace the need for humans; rather, it will open up even more exciting job opportunities in medicine that will harness the immense potential for critical thinking of the human brain.

While the deployment of these technologies is still in its infancy in Vietnam, the trajectory is set for exponential growth. This will be seen particularly as the market recovers from the restrictions imposed by the COVID-19 pandemic as well as a result of the government's intensive efforts to make Vietnam a high-income country by 2045. The future holds enormous promise in terms of attaining greater acceptability and practicality of AI and robotics in healthcare, thereby providing efficient data management and surgical accuracy. This advancement will require the close cooperation of public and private sectors, hitherto not known for collaboration, as well as a sincere commitment to overcome the logistical and financial challenges that may arise. As technological innovations are progressively adopted, not only will these enhance patient care experiences and improve outcomes but also pave the way for a more resilient and advanced healthcare system in Vietnam over the coming decades.
